# Factors influencing pre-hospital care time intervals in Iran: a qualitative study

**DOI:** 10.5249/jivr.v10i2.953

**Published:** 2018-07

**Authors:** Davoud Khorasani-Zavareh, Reza Mohammadi, Katarina Bohm

**Affiliations:** ^*a*^Safety Promotion and Injury Prevention Research Center, Shahid Beheshti University of Medical Sciences, Tehran, Iran.; ^*b*^Department of Health in Disaster and Emergencies, School of Health, Safety and Environment, Shahid Beheshti Uni-versity of Medical Sciences, Tehran, Iran.; ^*c*^Department of Clinical Sciences and Education, Södersjukhuset (KI SÖS), Karolinska Institutet, Stockholm, Sweden.; ^*d*^Department of Neurobiology, Care Sciences and Society, Division of Family Medicine, Karolinska Institutet, Huddinge, Sweden.

**Keywords:** Pre-hospital emergency management, Response time interval, Road traffic crash

## Abstract

**Background:**

Pre-hospital time management provides better access to victims of road traffic crashes (RTCs) and can help minimize preventable deaths, injuries and disabilities. While most studies have been focused on measuring various time intervals in the pre-hospital phase, to our best knowledge there is no study exploring the barriers and facilitators that affects these various intervals qualitatively. The present study aimed to explore factors affecting various time intervals relating to road traffic incidents in the pre-hospital phase and provides suggestions for improvements in Iran.

**Methods:**

The study was conducted during 2013-2014 at both the national and local level in Iran. Overall, 18 face-to-face interviews with emergency medical services (EMS) personnel were used for data collection. Qualitative content analysis was employed to analyze the data.

**Results:**

The most important barriers in relation to pre-hospital intervals were related to the manner of cooperation by members of the public with the EMS and their involvement at the crash scene, as well as to pre-hospital system factors, including the number and location of EMS facilities, type and number of ambulances and manpower. These factors usually affect how rapidly the EMS can arrive at the scene of the crash and how quickly victims can be transferred to hospital. These two categories have six main themes: notification interval; activation interval; response interval; on-scene interval; transport interval; and delivery interval.

**Conclusions:**

Despite more focus on physical resources, cooperation from members of the public needs to be taken in account in order to achieve better pre-hospital management of the various intervals, possibly through the use of public education campaigns.

## Introduction

Pre-hospital management is unsatisfactory in many parts of the world and particularly in low- and middle-income countries (LMICs).^1,2^ In addition, the majority of trauma deaths occur in the pre-hospital phase.^3^ Pre-hospital emergency care should be organized as an integral component of health care systems in terms of physical and manpower resources.^4,5^ Well-planned and coordinated pre-hospital systems are a necessity as they can help to significantly reduce preventable deaths; injuries and their consequences; ^3,6,7^ as well as reduce long-term disabilities and psychologically related trauma caused by road traffic injuries (RTIs). ^8^ Among the various components, rapid arrival of the emergency medical services (EMS) at the crash scene and proper victim transportation by trained personnel can reduce injury severity and the number of preventable deaths either at the crash scene or at the hospital.3 Victim transportation faces problems related to not only transportation itself but also the manner in which laypeople evacuate victims.^9^ Improvements in pre-hospital emergency management can increase the effectiveness of rescue services, shorten response times to incidents and improve road traffic crash (RTC) victim transportation to medical facilities. ^10^

Victim transportation, especially in LMICs, if available, is usually provided by untrained people; e.g., relatives, taxi drivers, commercial drivers, truck drivers, and even police officers.^1,2,11^ Studies have shown that the inadequacy of public health infrastructure and poor access to health services are important reasons for the high number of RTIs and/or their severity. ^12^ These problems are exacerbated in LMICs.^13^

In Iran, a middle-income country, the proportion of victim transportation carried out by the EMS increased sharply after a dramatic improvement in post-crash management^10,14^ and increased from 14% to more than 80% during a 12-year period starting in 2003. Improvements included a greater number of ambulances and ambulance dispatch sites, the provision of better equipment, more staff, and educational plans for emergency team members, and the introduction of helicopter and motorcycle ambulances in the EMS.^15,16^ All these improvements were aimed at reducing pre-hospital time intervals, with more focus on the response time interval. However there is still problem in saving lives of RTC victims and it is not clear which factors affect these intervals.

Research indicates that the first 60 minutes after injury occurrence - referred to as the “golden hour"- is one of the most crucial time intervals when it comes to saving lives. ^16^ This “golden hour” consists of various intervals: a notification interval; activation interval; response interval; on-scene interval; and transport interval.^16^ While a few studies in Iran have focused on measuring these intervals, there is no study to the best of our knowledge aimed at identifying barriers related to various intervals and how they can be reduced. ^3^ Accordingly, the present study was designed to explore factors affecting various intervals in the pre-hospital phase of road traffic injuries according to stakeholders’ perceptions at both national and local level in Iran. 

## Methods 

Qualitative content analysis based on Graneheim & Lundman was used to analyze the data in order to describe or delimit categories.^17^ The study was conducted during 2013-2014.

**Study participants**

Study participants were selected by means of purposeful sampling at both national (Tehran) and local level (Urmia City) in Iran. Participants comprised 14 EMS technicians, 1 physician and 3 EMS dispatch center personnel, aged from 22 to 35 years old with a wide range of work experience. Participants were selected from those willing to participate in the study, able to communicate with the interviewer, and with experience of working in the EMS.

**Data collection**

In-depth and semi-structured interviews were employed for data collection. Interviews started with general questions, gradually progressing to more specific ones. This kind of interview was appropriate because of the flexibility and depth of qualitative research.^18^

Each interview began with a broad question, e.g. “Could you explain your experience of factors affecting pre-hospital time intervals?”, “How do you feel these factors affect response intervals?” and “How about your experience regarding activation intervals?” Later questions in the interview focused on specific intervals. The time and place of the interviews were determined by participants in Tehran and Urmia, and the interviews took place at EMS centers.

Interviews were conducted individually and in Persian and recorded with the consent of participants. After completion, transcripts were produced verbatim. Each interview, depending upon participants’ conditions and mutual agreement, lasted between 40 and 70 minutes. The number of participants was determined based on the saturation principle,^2^which means the researcher decided when collected data was being repeated, new code was not being developed or existing codes were not being extended. The various time intervals, as shown inAppendix I, were also used as a general guide for data collection.

**Appendix I:**
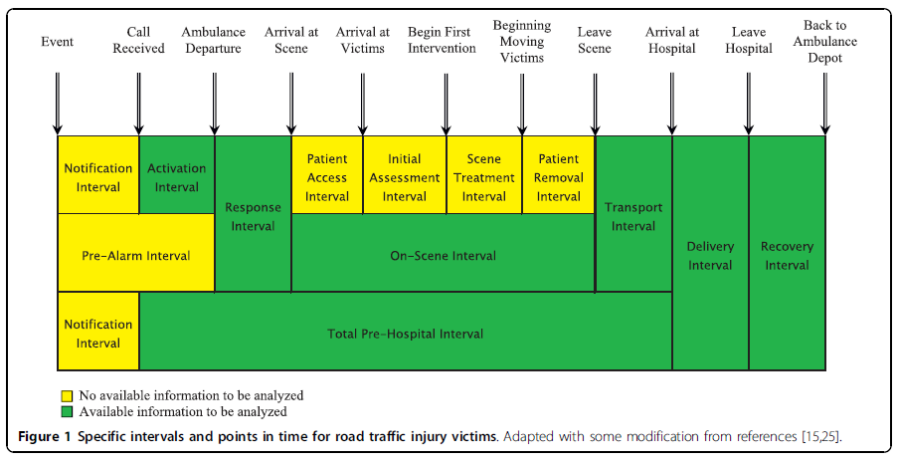
The notification interval is the time between injury occurrence and receipt of the call by the control facility of the emergency medical services; The activation interval is the time from receipt of the call by EMS facility control to departure of the ambulance from its depot; The response interval is the time from the ambulance departure to arrival on the crash scene; The on-scene interval is the time between ambulance arrival on the scene and departure from the scene. It consists of the patient access interval, initial assessment interval, scene treatment interval and patient removal interval; The transport interval is the time from departure of the ambulance from the scene to arrival at the hospital; The delivery interval is the time between arrival of the ambulance to the hospital and the delivery of the patients or victims to the emergency ward; The total pre-hospital time interval is the time between the initial call receipt by the EMS control facility and the ambulance’s arrival at the hospital; and the recovery time interval is the time between the emergency medical service personnel leaving the hospital and arriving back at the ambulance depot.

**Data analysis**

All interviews were transcribed verbatim and the transcripts of interviews with field notes and other documents were analyzed using qualitative content analysis.^19^ The analysis started by identifying meaning units as sentences that were essential to the participants’ experience and were extracted from the statements/transcript. The codes were compared based on differences and similarities and sorted into categories and then into sub-categories that were discussed within the research team; and appropriate categories were extracted from the data.

**Trustworthiness**

In order to ensure trustworthiness in this study, the first principal investigator had experience in the field of emergency medicine as well as pre-hospital management and has been working in the EMS for several years. This experience helped him to find and contact eligible key persons in order to select informed participants, and to allocate enough time for conducting interviews and the whole study. Moreover, triangulation by means of data collection including in-depth interviews, and field notes was employed to increase validity of the data. Researchers’ triangulation with different backgrounds of the research team members was also employed in order to include different perspectives during data analysis. Moreover, the principal investigator had presented extracted codes and initial categories, checked by two participants and peer-checked by one qualitative researcher.

**Ethical considerations**

First of all, permission was obtained by the Urmia University of Medical Sciences to conduct the study. The aim of the study was explained to the participants and their verbal or signed informed consent was obtained; data confidentiality was emphasized, and ethical principles were complied with regarding the recording of field notes. Moreover, it was emphasized that participants at each stage of the research had the right to withdraw from part or all of the study.

**Findings**

This study looked at the experience of stakeholders in relation to various intervals formulated in six main categories. These categories were: constructed notification interval; activation interval; response interval; on-scene interval; transport interval; and delivery interval. Two main issues were identified; namely a lack of cooperation from laypeople and problems relating to city infrastructure, in particular relating to traffic congestion. Definitions of the various intervals are presented in theAppendix I.

**Notification interval**

According to participants, different factors affect the notification interval. The first reason for a long notification interval is that members of the public initially want to rescue or release victims from trapped vehicles themselves instead of calling the EMS. Another very important factor, particularly in small cities, is the lack of resources at EMS dispatch centers. Usually, there are only two EMS staff members at each dispatch post and if they are called out on a mission, there is nobody to answer other calls. Telecommunication problems, including many radio and network black spots as well as too many emergency numbers are other important factors that result in confusion among laypeople when calling the EMS when a crash occurs. These telecommunication problems also affect EMS personnel, creating another significant obstacle to a rapid notification interval. The lack of a national emergency organization and a single three-digit number, as pointed out by EMS professionals, also indirectly affects the notification interval.

…In most rural areas, there is no full-time dispatch center to process incoming calls. When we are called out, there is no other EMS personnel on call to answer members of the public when they call…before doing anything untrained people want to help victims and rescue them, instead of calling EMS, particularly in rural areas, and after wasting time, they then remember that they should call EMS. This did waste time….

The other reason for long notification interval may be is a lack of skillful personnel in dispatch centers to help members of the public who call the EMS. A heavy workload at dispatch centers and a lot of calls from members of the public also increase the notification interval.

**Activation interval**

Based on the experience of EMS personnel, heavy workload, insufficient manpower, not enough ambulances and EMS centers, non-paramedic activities and a lack of motivation among some EMS personnel are important barriers that increase the activation interval. Moreover, a lack of enough time for rest for EMS personnel at work, a high number of call-outs, too much to do, insufficient personnel on each work-shift particularly in rural areas, a lack of well-established EMS centers, equipment and facilities all prolong the activation interval. Since there are usually only two EMS personnel at most of the 18 EMS posts, they have to do other non-EMS activities like providing food, which indirectly affects their main role as EMS staff. While the activation interval is affected by different problems in both small and big cities in Iran, it is particularly problematic in rural areas with low numbers of EMS personnel.

….in Urmia city, we need at least six personnel to serve a population of 700 000, but instead we have only two personnel on each shift with lots of work….there are a large number of call-outs with no rest…we are human and also need to eat and rest, what can we do when we have to work 24-hour shifts with no substitute …

**Response interval**

All participants stated that the response interval in Iran is considered to be the most important EMS performance interval, which is monitored by Ministry of Health and Medical Education (MOHME) as well as other organizations. All professionals, particularly at MOHME, are sensitive about this interval and they are always evaluating it. The most important barrier to a good response interval is urban infrastructure, which is particularly important in bigger cities and especially in Tehran as the capital city of Iran. Based on participants’ experience, a lack of an emergency lane for EMS vehicles, traffic congestion, a lack of cooperation from members of the public with EMS personnel, poor coordination among emergency organizations, no well-designed ambulance system particularly at inner-city facilities, shortcomings in EMS ambulances and at EMS facilities, a lack of good GPS maps are all highlighted as the most important barriers to short response intervals. Moreover, the current infrastructure in Iran is not well adapted to emergency situations. Concerning GPS, it is important to note that poor navigation of ambulances as well as a lack of good GPS maps are significant barriers in urban areas in Iran. This situation is exacerbated by lack of a well-coordinated national system.

….there is no well-established GPS system in Iran and we are confused many times when we are called out on a mission, we don’t know which way is the best and the nearest way to get to victims….

Furthermore, a lack of both knowledge about emergencies and cooperation from untrained persons in inner-city traffic areas is considered to be one significant barrier to shorter response times. As expressed by most participants, members of the public should cooperate with the emergency services and give way to them in traffic when they see an ambulance, even without its siren sounding. However; there is a lack of good cooperation in this respect. This results in log response intervals.

Member of the public should cooperate better with EMS, even when they are driving without their siren and flashing lights on….this lack of cooperation with us is a common problem.

**On-scene interval**

The on-scene interval consists of four sub-intervals: patient access interval; initial assessment interval; scene treatment interval; and patient removal interval. Overall, based on participants’ experience, there is a common belief among members of the public that the only important responsibility of the EMS is rapid evacuation of victims. Laypeople usually feel that removing victims from a crash site and quickly taking them to health centers are the best courses of action for the victims’ survival. This is more usual in small cities, where laypeople often insist that EMS personnel transport RTC victims from the crash scene to hospital and a health center as quickly as possible. According to participants, member of the public want to help victims and this is why they come to crash scenes and interfere with the duties of EMS personnel. The involvement of untrained people even results in Advance Life Support not being administered at crash scenes and EMS personnel not giving this care until the victim is in the ambulance. 

There is also a lack of knowledge among members of the public about victim management and what is better for the victim; victims should be moved properly by means of safe transportation. Untrained people usually focus on victims’ wailing and groaning, which triggers emotional behavior and prevents them from managing the victim in a safe and efficient manner. A lack of crash scene management is another factor that affects EMS personnel activities. The actions of untrained people affect all the four intervals listed above.

….people are usually in hurry at the crash scene and they want to move the victim to the ambulance and then to the hospital. They only want to move them, without paying attention to the importance of their immobilization and their care ….as a result of untrained people’s haste; some of victims suffer spinal cord injuries. In such cases, victims must be fixed initially and then moved to the ambulance with care….

**Transport and delivery interval**

The most important barriers to short transport intervals described by participants were: traffic jams and congestion in urban areas and a lack of cooperation of people with EMS ambulances, resulting in their late arrival to hospitals. Participants also pointed out that members of the public do not respect the ambulance siren and they don’t know what to do when an ambulance sounds its siren. Sometimes there is a lack of trust in ambulance personnel and people think that they turn their siren on only to get to work more quickly, which is not an emergency. This results in long transport intervals by ambulance. Lack of cooperation and trust were expressed as the major barriers in this situation. Regarding the recovery interval, approximately the same aspects were highlighted by participants. Accordingly, the most important barriers are congestion in urban areas and a lack of cooperation from members of the public cooperation with EMS ambulances.

Focusing on the delivery interval, EMS personnel also indicated that there is a lack of cooperation from hospital personnel at emergency department (A&E) with EMS personnel. The most important factor affecting the cooperation of A&E staff is also hospital overcrowding.

A&E personnel think that we cause them problems and are responsible for overcrowding … while in fact we are only doing our job and only transfer patients and victims to the nearest hospitals as instructed by the dispatch center. 

## Discussion

This study, the first of its kind, can clarify factors affecting functions of all pre-hospital time intervals in a middle-income country. Based on participants experience, the most important barriers in relation to pre-hospital time intervals were related to the interaction of untrained people and to city infrastructure, which commonly delay arrival at the accident scene or hospitals. These two important aspects should be improved in order to improve pre-hospital time interval management. Shortcomings in pre-hospital systems and resources were not emphasized.

Focusing on the notification interval, an inappropriate telecommunication system and many network black spots are important factors that affect it, something which has been highlighted in previous studies in Iran.^3^ There are reasons for this including large areas and limitations in radio communications. ^14^ Difficulties regarding the delivery of a well-developed wireless system also affect health and health systems in Iran. Moreover, a lack of knowledge among laypeople regarding the manner of their interaction when getting involved at crash scenes also increases this interval. ^10^ One study has already indicated that this interval is sometimes more than one hour and has a considerable effect on total pre-hospital intervals. ^20^ Currently, Iran has 39 different three-digit EMS numbers and members of the public are often unfamiliar with them. Further, members of the public can be confused after a crash as to what they should do. It is important to note that a previous study in Iran has also indicated that establishment of a single emergency number and single answering point would mostly solve this problem.^10^ A decision to establish such a single number and public safety answering service is currently being discussed in Iran, but it needs more support from Road Safety Commission as National Authority. It should also be reiterated that heavy workload, shortcomings in the EMS system and too few EMS personnel at each dispatch center result in delays when answering calls from members of the public.

Regarding the response interval, it is important to note that several factors affect it, including distribution and availability of resources; communication systems and transportation; the manner of public interaction in city areas; and infrastructure. Of these, the manner of public interaction seems to be the most important factor affecting response intervals.^10,14^ It seems that, due to major developments in the number of ambulances, personnel and EMS facilities, the problem cannot mainly be caused by the lack of resources. ^14^ It is instead due to a lack of cooperation by laypeople with the EMS. ^3,10^ However, regarding resources, findings from the current study do partly contradict a study by Mock et al ^4^ indicates that physical and human resources are more important than organization and administration. ^21^ In our study, however, the most important weak points in the trauma systems were caused by a lack of coordination and cooperation of untrained people. This does not detract from the fact that shortcomings in professional staff, the EMS system and the skill of dispatch sites are still significant obstacles to providing effective pre-hospital trauma services.^14^

Focusing on the on-scene interval, this interval is mainly related to the manner in which people interact at the crash scene, which may be because of a sense of hesitation and curiosity among laypeople.^10^ They want to help RTC victims, but this is sometimes in contrast with recommendations by the World Health Organization (WHO).^15^ According to participants, member of the public think that if they move victims from trapped vehicles and move them to hospitals, it is better for their survival. ^22^ The main problem with this situation is improper immobilization that may result spinal cord injury. WHO recommends that members of the public call the emergency services as their first action and then administer first aid to and provide security for victims at crash scenes. These actions have been already discussed in previous studies in Iran. ^3,23^ These recommendations are usually ignored, however, and laypeople intervene at crash scene. It is important to note that a previous study also indicate that the on-scene interval is mainly related to the skill of EMS personnel, while participants in the current study stated that this interval mainly relates to the manner in which people interact at the crash scene. This is because many activities have been taken place in EMS system in recent years ^22^ to improve the skills of EMS personnel by means of further training. This has resulted in better performance of EMS personnel and has improved their skills at crash scenes. Participants in this current study indicated that the interaction of laypeople at the crash scene usually results in victims being moved to the ambulance before they are stable. This results in EMS personnel only staying at the crash scene for a short time, which sometimes has a negative effect on the safety of RTC victims. It implies that one important measure for emergency organizations to take is to provide public education campaigns to instruct member of the public how they should interact in such circumstances. This measure should also be considered for other emergency situations, such as the fire and subsequent collapse of the Plasco building in Tehran. ^24^

It is important to note that the on-scene interval comprises four sub-intervals including: patient access interval; initial assessment interval; scene treatment interval; and patient removal interval. All these are affected by laypeople’s involvement at the crash scene. The patient access interval is one of the most problematic issues as it is the result of traffic congestion and laypeople’s involvement as bystanders. Public education campaigns should be considered to improve this situation. After the Plasco building collapse, such a campaign helped EMS members to solve the situation in a better way. EMS personnel prefer to move victims to ambulance and continue their treatment there to achieve a better outcome. Moreover, a long patient access interval is also related to the city infrastructure and the availability of hospitals as well as traffic congestion.

## Conclusion

The most important barriers to short pre-hospital intervals are the manner of laypeople cooperation with the EMS and their involvement at the crash scene. These usually prevent rapid arrival at the scene or at hospital. They also affect all intervals that could be improved by public education campaigns as well as by having better EMS equipment. Despite more focus on physical resources in the pre-hospital system, cooperation of laypeople needs to be taken into account in order to achieve better management of various intervals.
